# *Antheraea pernyi* Suppressor of Cytokine Signaling 2 Negatively Modulates the JAK/STAT Pathway to Attenuate Microbial Infection

**DOI:** 10.3390/ijms231810389

**Published:** 2022-09-08

**Authors:** Saima Kausar, Isma Gul, Ruochen Liu, Xiao-Xue Ke, Zhen Dong, Muhammad Nadeem Abbas, Hongjuan Cui

**Affiliations:** 1 State Key Laboratory of Silkworm Genome Biology, Key Laboratory of Sericultural Biology and Genetic Breeding, Ministry of Agriculture, Southwest University, Chongqing 400716, China; 2 Affiliation Cancer Center, Medical Research Institute, Southwest University, Chongqing 400716, China

**Keywords:** innate immunity, JAK/STAT pathway, antibacterial immunity, SOCS proteins

## Abstract

The Janus kinase (JAK) signal transducer and activator of transcription (STAT) pathway has been shown to govern various physiological processes, including immune responses, hematopoiesis, cell growth, and differentiation. Recent studies show that suppressors of cytokine signaling (SOCS) proteins attenuate JAK-STAT signaling in mammals; however, their functions are less clear in lepidopteran insects. Here, we report a full-length sequence of SOCS-2 from the Chinese oak silkworm *Antheraea pernyi* (designated as *ApSOCS-2*) and study its biological role in immune responses via the JAK-STAT pathway. *ApSOCS-2* expression was high in the fat bodies and hemocytes of *A. pernyi* fifth instar larvae. After pathogen infection with nucleopolyhedrovirus, *Beauveria bassiana, Escherichia coli*, and *Microccus luteus*, *ApSOCS-2* mRNA was strongly increased compared to the control group. To elucidate the possible involvement in innate immunity, we measured antimicrobial peptide genes expression profiles in the fat body of *A. pernyi*. In contrast, recombinant ApSOCS-2 protein administration significantly reduced the AMPs transcription, while the depletion of *ApSOCS-2* by RNAi increased their expression. Furthermore, we observed higher antibacterial activity and lower bacterial replication in *dsApSOCS-2*-treated larvae. The *ApSOCS-2* transcription level was reduced in STAT depleted *A. pernyi* larvae challenged by *M. luteus*. The *ApSOCS-2* RNAi data sets were also subjected to transcriptomic analysis, which suggests that *ApSOCS-2* is a key regulator of immune function. Taken together, our data suggest that *ApSOCS-2* is required for the negative regulation of AMPs transcripts via the JAK-STAT pathway in the insect.

## 1. Introduction

The innate immune system of insects is similar to that of vertebrates but not as advanced as the acquired immune system [1,2]. The innate immune system comprises cellular and humoral immunity, and the latter plays a critical role in insect immune defense [3,4]. Humoral immunity is devoid of antibodies and complement systems; however, antimicrobial peptides and other bioactive factors are produced in the fat body or other immune tissues and then secreted into hemolymph for the clearance of invaded pathogens [5]. Generally, the innate immune response contains three biological processes: recognition of pathogens, signal transmission and transcription of immune-associated genes [6,7,8], and regulation by signaling pathways [2]. 

Cytokines (e.g., interferon and interleukins) play a critical biological role in hematopoiesis, cell proliferation, differentiation, and immunity [9]. These proteins are important mediators of cell-to-cell communication. They are secreted by cells upon environmental stimuli to forward biological information to neighboring cells bearing the suitable receptor on their surface. The cell surface message is immediately transferred to the nucleus by different signaling cascades. The most important one regarding cytokines is the JAK-STAT pathway. However, it seems evident that cytokine actions must be stringently controlled in duration and magnitude. Indeed, abnormal cytokine signaling has been associated with different diseases, such as disorders in hematopoiesis, autoimmune diseases, and many types of cancer. Several key modulatory proteins, including the protein family of suppressors of cytokine signaling (SOCS), protein inhibitors of activated STATs (PIAS), and the Src-homology 2 (SH2)-comprising protein tyrosine phosphatases (SHPs), regulate cytokine responses [10,11].

The crucial modulators of cytokine signaling, SOCS proteins, are rapidly induced upon JAK-STAT signaling by activated STATs to negatively control cytokine signaling via a classical feedback loop [12]. The SOCS proteins contain a conserved central SH2 domain, an amino-terminal domain of variable sequence and length, and a carboxy-terminal 40 amino-acid region called the SOCS box. The SOCS can interact with a series of signaling intermediates via the binding of their SH2 domain to phosphorylated tyrosine residues, especially those on JAKs and cytokine receptors, leading to the blockade of the signal [11,12]. Due to the SOCS box, they can act as ubiquitin ligases for associated proteins and target them for proteasomal degradation. Within the E3 ligase complex composed of Rbx1/2, cullin5, elonginBC, and SOCS2, SOCS2 thus constitutes the substrate recognizing component [13,14,15]. The function of each member of the SOCS proteins is somewhat difficult to analyze as these proteins may reciprocally control each other due to their ubiquitin E3 ligase activity [16,17]. Therefore, their biological roles in different functions have to be interpreted.

The economically important insect, *A. pernyi*, is widely used as a raw silk producer and a model species. However, information about the innate immune system and associated factors is limited, particularly the SOCS/CIS gene family and their regulatory role in AMPs production [18]. This is in contrast to the studies that have been performed on SOCS proteins and the associated JAK-STAT pathway in vertebrates and invertebrates. For example, the induction of SOCS-2 involving JAK-STAT signaling has been described in vertebrates [19,20]. In addition, intracellular signaling associated with the JAK-STAT pathway and the AMPs responses has been explored in different invertebrate species [21,22,23,24]. In the present study, we report for the first time the identification and cloning of the full-length cDNA sequence of the Chinese oak silkworm, *A. pernyi* (*ApSOCS-2*), and analyzed its sequence features and phylogenetic relationships at the amino acid level. The spatial and temporal expression levels of *ApSOCS-2* mRNA and its responses to viral (ApNPV), fungal (*B. bassiana*), and bacterial (*M. luteus* and *E. coli*) challenges were characterized at different time points. We used recombinant protein and RNAi to determine the involvement of ApSOCS-2 in the AMP genes transcription and suppression of bacterial infection. Furthermore, we conducted RNA sequencing after RNAi to explore the regulatory network of the *ApSOCS-2* gene. Our study advances an understanding of the *ApSOCS-2* regulatory signaling pathway in the immune response of *A. pernyi* via the regulation of transcription of AMP genes.

## 2. Results

### 2.1. Sequence and Phylogenetic Analysis of ApSOCS-2

**Figure 1:**
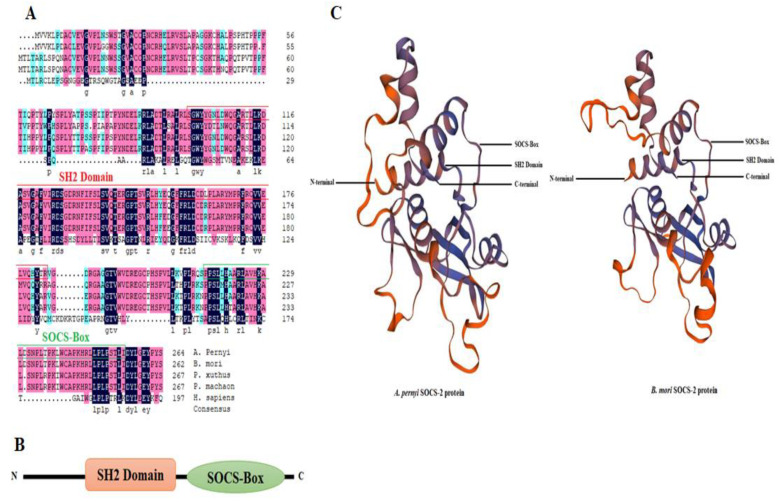
Multiple sequence alignment and tertiary structure of ApSOCS-2 protein. (**A**) Sequence alignment of ApSOCS-2 protein with those of other SOCS/CIS family proteins from *Bombyx mori* (NM_001256992.1), *Papilio xuthus* (KPJ02665), *Papilio machaon* (KPJ08265), and *Homo sapiens* (NP_001257396). The conserved domains are represented by red and green boxes in the sequence alignment. (**B**) Conserved domains of ApSOCS-2 protein: SH2 Domain and SOCS-Box of *Antheraea pernyi.* (**C**) The prediction of SOCS-2 protein tertiary structure of *A. pernyi* using SWISS-MODEL program. The SH2, SOCS-box domains, N and C terminals in the protein structures are labeled.

We used the *A. pernyi* RNA sequence data to retrieve the *ApSOCS-2* sequence and then confirmed it by PCR amplification. The full-length retrieved sequence of *ApSOCS-2* was 4048 bp, the transcription start site was recognized at 932 bp upstream of the translation initiation codon (ATG), and the poly-A was identified at 2302 bp downstream of the translation stop codon (TAA). The open reading frame (ORF) sequence comprises 798 nucleotides encoded by 265 amino acid residues (Figure S1). The calculated molecular weight of ApSOCS-2 protein was 29.49 kDa with an isoelectric point (pI) of 8.97. Conserved domain analysis revealed that ApSOCS-2 contains a SH2 domain (86 amino acids) at the downstream of the N-terminus and a SOCS-box domain (77 amino acids) at the upstream of the C-terminus, similar to that of the SOCS proteins of other animals (Figure S1 and Figure 1A–C). These domains are evolutionarily conserved in invertebrate and vertebrate species. The SOCS-box is critical for the recruitment of the E3 ubiquitin ligase scaffold Cullin 5, which then catalyzes the ubiquitination of phosphorylated signaling intermediates bound to the SH2 domain [11,25,26]. These substrates are presumed to include both the JAKs and the cytokine receptors to which they are bound.

**Figure 2:**
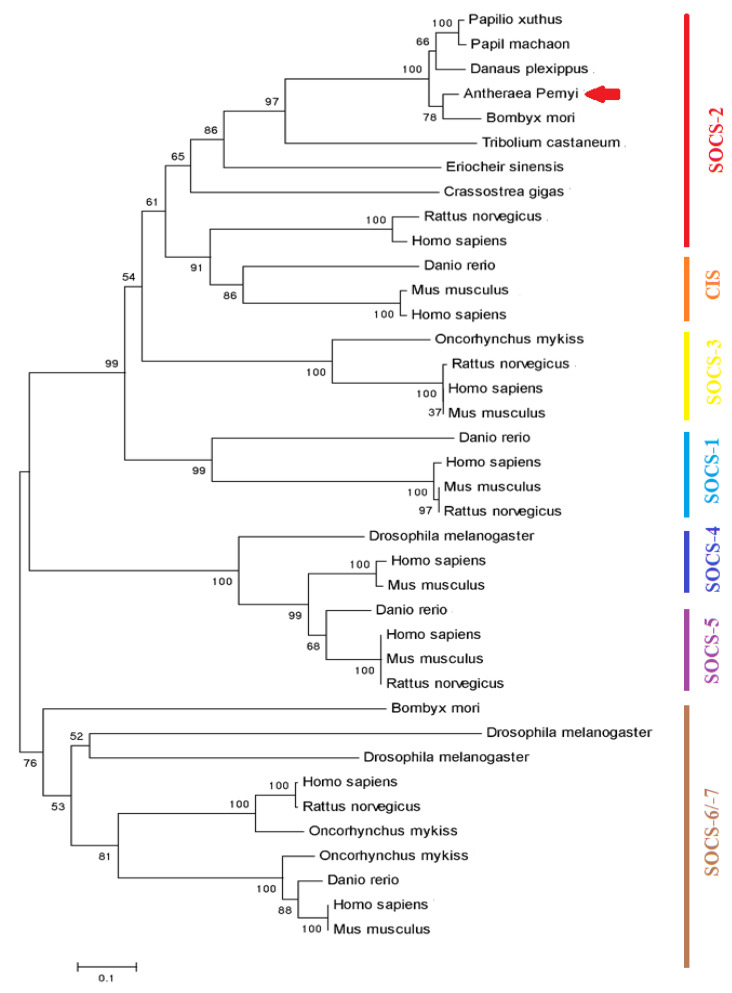
Phylogenetic tree of ApSOCS-2. The neighbor-joining phylogenetic tree of ApSOCS-2 with other orthologous proteins from invertebrate and vertebrate species with a bootstrap of 1000 replicates. An arrowhead shows ApSOCS-2.

A blastp search of the ApSOCS-2 amino acid sequence carried out against the NCBI database retrieved many other SOCS proteins exhibiting homology to SOCS-2 proteins in invertebrates and vertebrates. Multiple sequence alignment analysis of ApSOCS-2 with other organism homologs displayed that the SH2 and SOCS-box domains were highly conserved in the organisms, and ApSOCS-2 had the highest similarity to other lepidopteran homologs (Figure 1A). A phylogenetic tree was constructed using SOCS amino acid sequences from 37 representative invertebrate and vertebrate species (Figure 2). The results displayed the distribution of the SOCS proteins into five major groups, comprising CIS, SOCS-2, SOCS-3, SOCS-4, SOCS-5, and SOCS-6/-7. ApSOCS-2 was first grouped with its close relative, *B. mori* SOCS-2 protein, and then with the members of this insect group, comprising *Danaus plixuppus*, *Papil machaon*, and *Papilio xuthus*. Furthermore, although SOCS-2 proteins of vertebrates were clustered together based on their sequence similarity; however, they formed two major clades, one for invertebrate species and one for vertebrate species.

### 2.2. Protein Expression and Western Blot Analysis

**Figure 3:**
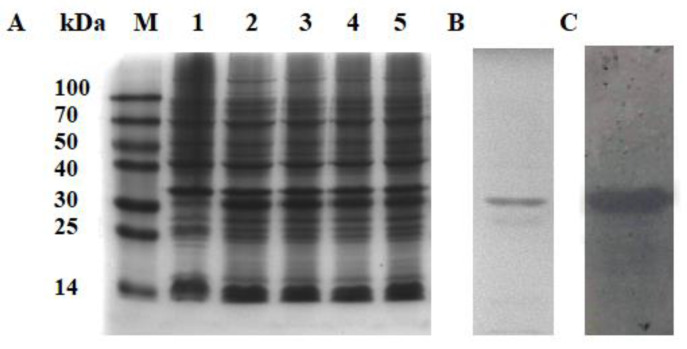
Recombinant protein expression and purification of ApSOCS-2. (**A**) SDS-PAGE analysis of *A. pernyi* SOCS-2 protein expression in *E. coli* induced by IPTG. M: molecular weight marker; lane 1, no IPTG treatment; lane 2, 0.3 mM IPTG treatment; lane 3, 0.5 mM IPTG treatment; line 4, 0.7 mM IPTG treatment; lane 5, 1 mM IPTG treatment. (**B**) SDS-PAGE analysis of recombinant ApSOCS-2 protein purified by affinity chromatography. (**C**) Western blot analysis of extracted ApSOCS-2 protein.

A thicker protein band with an approximate molecular weight of 29.49 kDa was observed in the protein extracts from *E. coli* induced by IPTG and subjected to SDS-PAGE, which was roughly consistent with the predicted molecular weight of ApSOCS-2 (Figure 3A). A purified ApSOCS-2 His-tag fusion protein was obtained via affinity chromatography, with a protein band corresponding to the predicted size of 29.49 kDa observed (Figure 3B). The western blotting analysis of the recombinant proteins, which used an anti-His-tag antibody, further confirmed a consensus protein of 29.49 kDa in size (Figure 3C). The purified recombinant ApSOCS-2 protein was then used to evaluate its biological role in vivo.

### 2.3. Spatial Expression of ApSOCS-2 Transcripts

**Figure 4:**
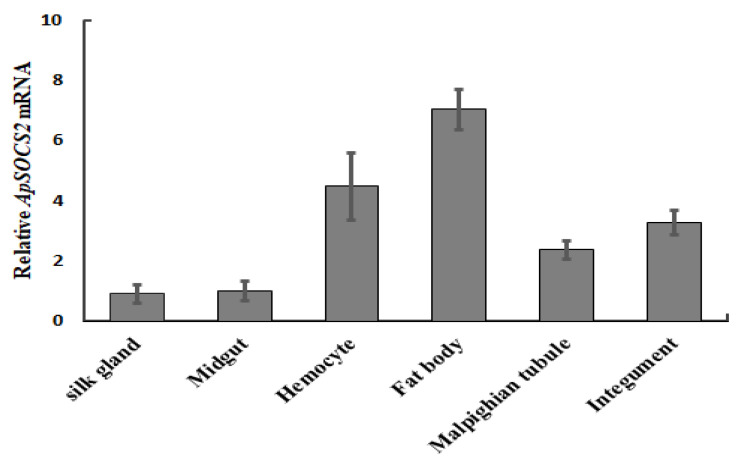
Expression profile of *ApSOCS-2* in various tissues of *A. pernyi* normalized to 18*S* and actin-1 (Actin-1 normalized: Figure S2). The mRNA expression profile of *ApSOCS-2* in various tissues of the *A. pernyi* was determined using qRT-PCR. The values are represented as mean ± S.E.

We analyzed the expression profiles of *ApSOCS-2* mRNA in different tissues (silk gland, midgut, hemocytes, fat body, Malpighian tubules, and integuments) of the Chinese oak silkworm, *A. pernyi,* using qRT-PCR. The expression of *ApSOCS-2* mRNA was observed in all the tested tissues of the insect but was more pronounced in the fat bodies and hemocytes, whereas it was lowest in the silk gland and midgut (Figure 4).

### 2.4. Microbial Challenge-Induced Expression of ApSOCS-2 Transcripts

**Figure 5:**
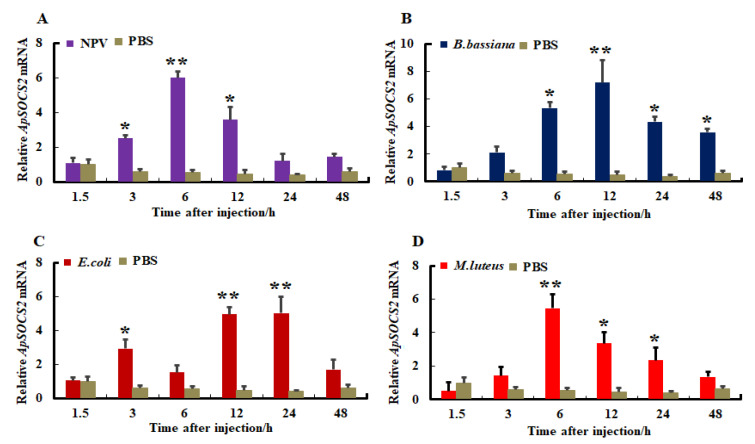
The mRNA expression levels of *ApSOCS-2* in fat bodies following microbial challenge normalized to 18*S* and actin-1 (Actin-1 normalized: Figure S3). Following injection of (**A**): ApNPV, (**B**): *B. bassiana*, (**C**): *E. coli*, (**D**): *M. luteus*, the mRNA expression levels of *ApSOCS-2* were analyzed using qRT-PCR. Data of triplicate experiments (n = 3) are represented with mean ± S.E. Asterisks indicate significant differences (* *p* < 0.05, ** *p* < 0.01).

**Figure 6:**
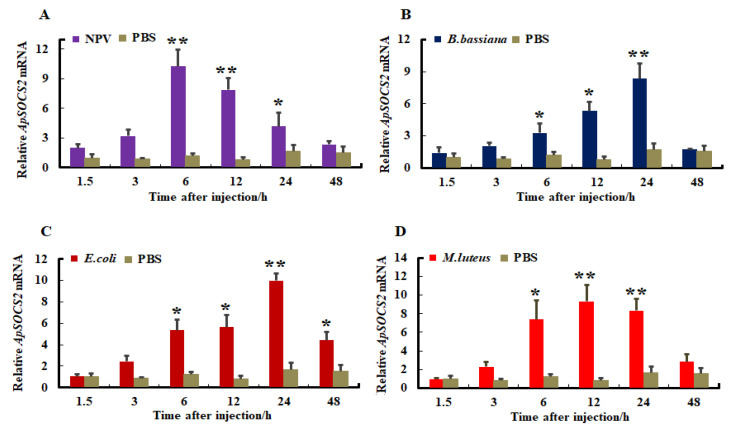
The mRNA expression levels of *ApSOCS-2* in hemocytes following microbial challenge normalized to 18*S* and actin-1 (Actin-1 normalized: Figure S4). Following injection of (**A**): ApNPV, (**B**): *B. bassiana*, (**C**): *E. coli*, (**D**): *M. luteus*, the mRNA expression levels of *ApSOCS-2* were analyzed using qRT-PCR. Data of triplicate experiments (n = 3) are represented with mean ± S.E. Asterisks indicate significant differences (* *p* < 0.05, ** *p* < 0.01).

The changes in expression profiles of *ApSOCS-2* mRNA were investigated in response to immune challenges with the ApNPV, Gram-positive bacteria *M. luteus*, Gram-negative bacteria *E. coli*, and the fungus *B. bassiana*. After the microbial challenges, fat body and hemocyte were sampled and used to synthesize cDNA, which was used as a template. The expression of *ApSOCS-2* mRNA levels differed substantially in hemocytes and fat bodies after being challenged with pathogens, and their mRNA expression further depended on the type of microbial pathogen. The viral, bacterial, and fungal pathogens remarkably stimulated the expression of *ApSOCS-2* mRNA in the tested tissues, but both the mRNA level and the time of maximum mRNA of *ApSOCS-2* differed among them (Figure 5 and Figure 6). *ApSOCS-2* showed maximum expression in fat bodies after 6 h of ApNPV and *M. luteus* infection, whereas this occurred at 12 and 24 h after *B. bassiana* and *E. coli* infection, respectively (Figure 5A–D). While in hemocytes, approximately similar expression patterns were observed as the maximum level of *ApSOCS-2* mRNA peaked at critical time points (12 and 24 h). It was highest at 24 h following the *B. bassiana* and *E. coli* challenges, whereas it peaked at 6 h and 12 h after ApNPV and *M. luteus* treatment, respectively (Figure 6A–D). The induction profiles in the microbial pathogen challenge studies suggest a putative role for *ApSOCS-2* in the immune response.

### 2.5. ApSOCS-2 In Vivo Knockdown Reduced Bacterial Replication

**Figure 7:**
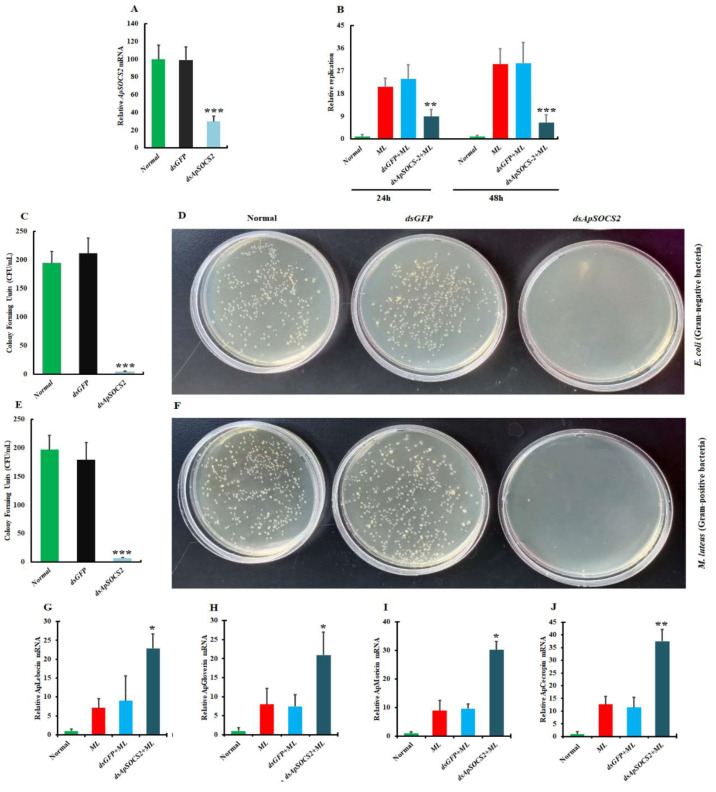
Loss-of-function analysis of *ApSOCS-2* on bacterial survival and antimicrobial peptides production normalized to 18*S* and actin-1 (Actin-1 normalized: Figure S5). (**A**): *dsApSOCS-2* injection strongly reduced the production of *ApSOCS-2* in hemocytes; (**B**): qPCR replication rate analysis exhibited that treatment of *A. pernyi* larvae with *dsApSOCS-2* decreased the replication rate of *M. luteus*. The plasma antibacterial activity was increased in the *ApSOCS-2* depletion (**C**–**F**). Plasma was sampled from *A. pernyi* treated with *ApSOCS-2* dsRNA or GFP dsRNA. Equal volumes (10 μL) of plasma and bacterial suspension were incubated at 25 °C for 1 h. The bacteria (*M. luteus*) was injected into *dsApSOCS-2* treated *A. pernyi* larvae, and mRNA expression levels of (**G**) lebocin, (**H**) gloverin, (**I**) moricin, and (**J**) cecropin were measured by qRT-PCR. Normal: untreated sample; ML: *M. luteus*-treated; *dsGFP* + ML: treated with both *dsGFP* and *M. luteus*; and *dsApSOCS-2* + ML: treated with both *dsApSOCS-2* and *M. luteus*. Asterisks indicate significant differences (* *p* < 0.05, ** *p* < 0.01, *** *p* < 0.001).

To determine whether *ApSOCS-2* is involved in conferring innate immune responses against Gram positive and Gram negative bacterial challenges, we produced double-stranded RNA against *ApSOCS-2* and knocked it down in *A. pernyi* whole-larvae. Following confirmation of efficient depletion, we evaluated bacterial replication after Gram positive (*M. luteus*) treatment by a qPCR assay. We synthesized a dsRNA for the *ApSOCS-2* gene or the GFP sequence to use as a control in the bacterial replication. We confirmed efficient suppression of *ApSOCS-2* (Figure 7A), then challenged *ApSOCS-2* knockdown *A. pernyi* larvae with *M. luteus* or *E. coli*, and subsequently analyzed the bacterial replications in *A. pernyi* larvae. The *dsGFP*-injected *A. pernyi* larvae were considered for comparison for bacterial replication. Our results revealed that bacterial (*M. luteus*) replications were significantly reduced compared to control groups (Figure 7B).

### 2.6. Effect of ApSOCS-2 Knockdown on Bacterial Clearance

To determine the function of the *ApSOCS-2* gene in the *A. pernyi*-pathogenic bacteria interactions, we depleted *ApSOCS-2* on the third day of fifth instar larvae (as described above) and analyzed the antibacterial activity of *ApSOCS-2* RNAi treated plasma. After incubation with *M. luteus* or *E. coli* with the plasma of *ApSOCS-2* silenced larvae, the survival rate of the bacteria was strongly reduced compared with the control groups (Figure 7C–F), suggesting that the *ApSOCS-2* suppression improves the clearance of bacteria.

### 2.7. ApSOCS-2 Regulates the Production of AMPs

**Figure 8:**
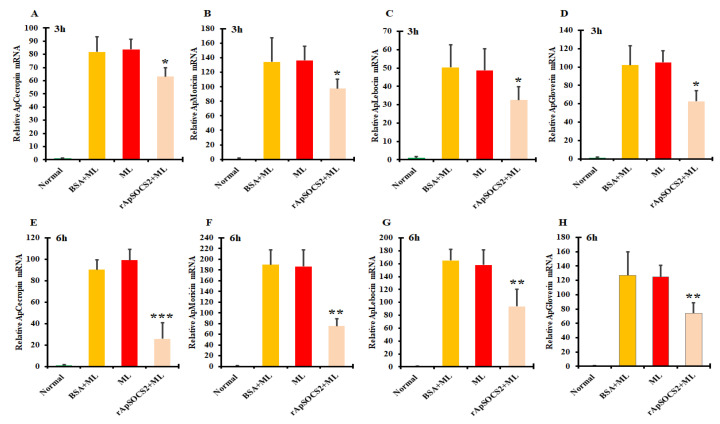
Relative mRNA expression levels of the pertinent AMPs, namely cecropin, moricin, lebocin, and gloverin (**A**–**D**) at 3 h after the recombinant ApSOCS-2 protein injection and (**E**–**H**) at 6 h after the recombinant ApSOCS-2 protein injection normalized to 18*S* and actin-1 (Actin-1 normalized: Figure S6). Normal: untreated; BSA + ML: treated with bovine serum albumin and *M. luteus*; ML: *M. luteus*-treated; and recombinant ApSOCS-2 + ML: treated with both recombinant ApSOCS-2 protein and *M. luteus*. Asterisks indicate significant differences (* *p* < 0.05, ** *p* < 0.01, *** *p* < 0.001).

To analyze the involvement of *ApSOCS-2* in humoral immunity, we measured the mRNA levels of four AMPs of *A. pernyi* following microbial challenge in vivo *ApSOCS-2* suppressed larvae by qRT-PCR. The expression of AMPs was compared between the *ApSOCS-2* silenced or GFP control groups. *A. pernyi* AMPs such as cecropin, moricin, lebocin, and gloverin were considered for the investigations. After confirmation of *ApSOCS-2* in vivo silence (Figure 7A), we treated larvae with Gram positive bacteria or PBS. After microbial administration, AMPs mRNA expression was measured. We found that in all of the AMPs analyzed, the expression levels were strongly increased at the tested time points compared to *dsGFP*-injected *A. pernyi* larvae (Figure 7G–J). These observations were confirmed by evaluating the AMPs transcription levels following the administration of recombinant ApSOCS-2 protein and *M. luteus*. The results revealed that the transcription levels of all tested AMPs (cecropin, moricin, lebocin, and gloverin) were significantly downregulated in the recombinant ApSOCS-2/*M. luteus-*treated sample compared with the control groups (Figure 8A–H).

### 2.8. ApStat Modulates ApSOCS-2 Production in A. pernyi Challenged with M. luteus

**Figure 9:**
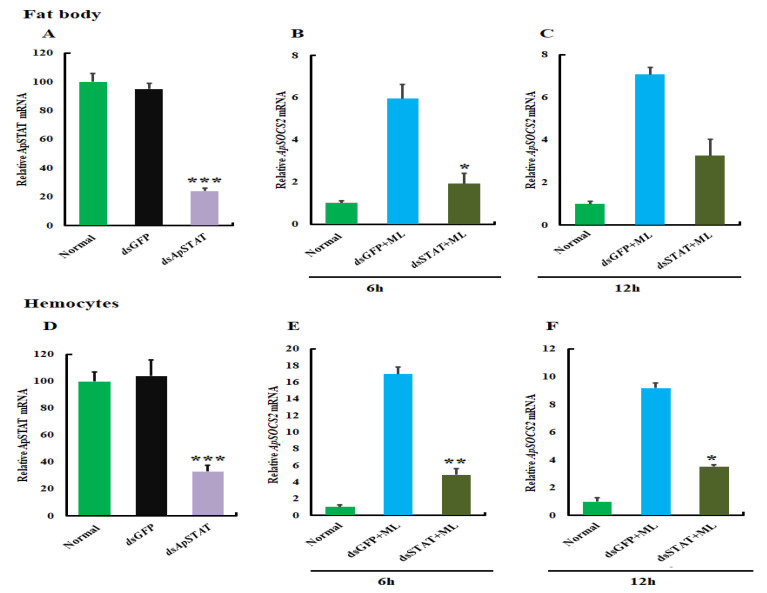
*ApStat* regulates *ApSOCS-2* expression in silkworm challenged with *M. luteus* at 6 and 12 h normalized to 18*S* and actin-1 (Actin-1 normalized: Figure S7). The efficiency of *ApStat*-RNAi in fat bodies (**A**) and hemocytes (**D**). (**C**,**D**) After *dsApStat* injection, the *ApSOCS-2* expression in fat bodies of silkworm challenged with *M. luteus* at 6 (**B**) and 12 h (**C**) was detected by qRT-PCR in fat bodies. Following *dsApStat* injection, the *ApSOCS-2* expression in hemocytes of silkworm challenged with *M. luteus* at 6 (**E**) and 12 h (**F**) was detected by qRT-PCR. The GFP-RNAi was used as the control. Asterisks indicate significant differences (* *p* < 0.05, ** *p* < 0.01, *** *p* < 0.001).

To analyze whether *ApStat* modulates *ApSOCS-2* expression, we down-regulated *ApStat* and then analyzed its effect following bacterial challenge. After *dsApStat* injection at 24 h, *ApStat* was depleted in fat bodies and hemocytes (Figure 9A,D). *ApSOCS2* mRNA expression was remarkably reduced in fat bodies (Figure 9B,C) and hemocytes (Figure 9E,F) at different time points (after 6 and 12 h) in the experimental group. The results suggest that *ApStat* regulates *ApSOCS-2* expression in insects.

### 2.9. Effects of ApSOCS-2 Suppression on the Gene Expression Patterns of A. pernyi

**Figure 10:**
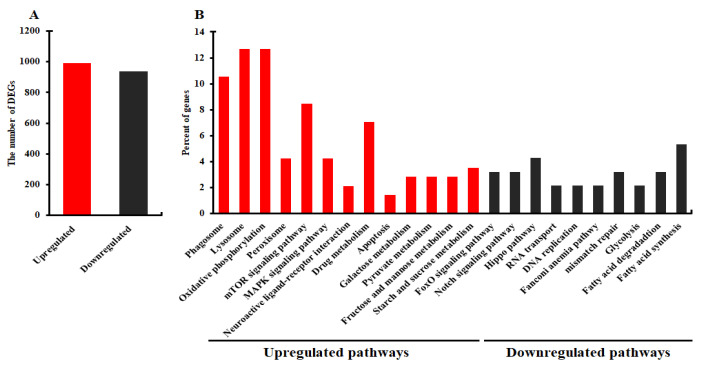
Transcriptome analysis following knockdown of *ApSOCS-2* in *A. pernyi*. (**A**): the number of DEGs in *dsApSOCS-2* and *M. luteus* treated larvae compared to *dsGFP* and *M. luteus* challenged group. (**B**): KEGG pathway enrichment analysis of differentially expressed genes in the transcriptome following *dsApSOCS-2* and *M. luteus* in *A. pernyi*.

To further understand the molecular mechanism by which *ApSOCS-2* is involved in the innate immunity of *A. pernyi*, we performed RNA sequencing using three replicates and analyzed the modifications in the expression patterns of genes in the fat bodies following *ApSOCS-2* depletion and bacterial challenge. The statistics of the transcriptome sequence data is shown in Table S3, indicating that quality and mapped rate of the sequence data are sufficiently high. The de novo transcriptome assembly used for the mapping consists of 42,847 contigs with total bases of 45,802,389 bp (Supplementary Materials Table S3). The DEGs analysis revealed that a total of 991 DEGs were upregulated, and 938 DEGs were downregulated after *ApSOCS-2* knockdown and bacterial injection (Figure 10A). The sequencing data and related information are represented in Supplementary Materials Files S1 and S2. The KEGG Pathway enrichment analysis of DEGs exhibited that the upregulated DEGs were mainly enriched in terms of the phagosome, lysosome, oxidative phosphorylation, peroxisome mTOR signaling pathway, neuroactive ligand-receptor interaction, drug metabolism, MAPK signaling pathway, and so on. However, the downregulated DEGs were mainly referred to as fatty acid synthesis, Fanconi anemia pathway, Hippo pathway, DNA replication, and so on (Figure 10B).

**Figure 11:**
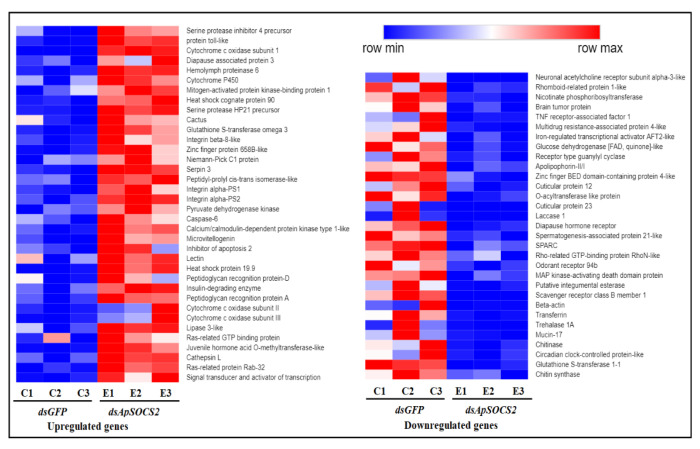
Cluster analysis of differentially expressed genes in the transcriptome following *ApSOCS-2* suppression and bacterial challenge in *A. pernyi*. Logarithmic fold alteration of *dsApSOCS-2* + *M. luteus* versus *dsGFP* + *M. luteus* is presented in the heat map. C1, C2, C3 represent the control group, and E1, E2, E3 indicate the experimental group, each with three replicates. Row min.: row minimum, and row max.: row maximum.

The expression analysis of DEGs revealed that among these DEGs, several genes encoding Diapause associated protein 3, Cytochrome P450, Heat shock cognate protein 90, and integrins, among others, were significantly upregulated. The expression levels of various important signaling pathways associated with, including signal transducer and activator of transcription, mitogen-activated protein kinase-binding protein 1, protein toll-like, serine protease inhibitor 4 precursors, juvenile hormone acid O-methyltransferase-like, and peptidoglycan recognition protein-D were strongly induced following *ApSOCS-2* knockdown. The identified DEGs may be involved in *ApSOCS-2* modulation in the innate immunity of *A. pernyi*. In contrast, *ApSOCS-2* suppression resulted in the remarkable downregulation of the circadian clock-controlled protein-like, cuticular protein, odorant receptor 94b, chitinase, scavenger receptor class B member 1, mucin-17, brain tumor protein, and others (Figure 11).

## 3. Discussion

In the present study, we identified a full-length sequence of the *ApSOCS-2* gene in the lepidopteran insect, *A. pernyi*, employing bioinformatics analysis. The ApSOCS-2 amino acid sequence was well within the range of SOCS proteins reported in other insect species (Figure 2). Like vertebrates and insect SOCS-2 homologs, it exhibited a completely conserved SH2 domain and SOCS box. In animals, the SH2 domain of SOCS-2 protein has been reported to be crucial for binding to phosphorylated residues, particularly those JAKs and cytokine receptors, leading to the block or suppression of the signal [12]. Thus, it can be speculated that ApSOCS-2 binds and forms a complex with JAKs. This interaction could subsequently block JAK-STAT signaling, therefore negatively regulating immune responses. Similar research has been attempted in various invertebrate species to understand the host defense molecular mechanism against pathogens [22,23]. Altogether, we have identified the SOCS-2 gene from *A. pernyi* and molecularly characterized it in detail. Since limited research has been carried out on the functional characterization of SOCS-2 homologs in regulating AMPs via JAK-STAT signaling in insect species, this study will help to advance our understanding of innate immunity among insects.

This study revealed that the transcription levels of *ApSOCS-2* were increased at 6–12 h in immune tissues (fat bodies and hemocytes) after ApNPV, *B. bassiana*, *E. coli*, or *M. luteus* challenge compared to the control group. The SOCS protein upregulation in response to viral, fungal, and bacterial pathogens is not entirely new; this phenomenon has been described in various insect and invertebrate species. For example, in *T. molitor*, three SOCS proteins (SOCS-5, -6, and -7) are identified and shown to be induced by the fungal pathogen [27]. The expression of the SOCS-2 and SOCS-6 genes increased in *B. mori* after infection by viral, bacterial, and fungal pathogens [23,24]. These expression profiles have also been described in some other insects, e.g., *Anopheles culicifacies* [27]. Besides insects, upregulation of SOCSs in response to the immune challenge has also been shown in various invertebrates, including *Ruditapes philippinarum*, *Engleromyces sinensis*, *Pinctada fucata*, and *Haliotisdiscus discus* [21,28,29]. The remarkable increase of *ApSOCS-2* in response to viral, fungal, and bacterial pathogens indicates that *ApSOCS-2* may be involved in the innate immune responses of *A. pernyi*.

In mammals and *Drosophila*, SOCS has been shown to be involved in innate immunity via negatively regulating the JAK-STAT signaling pathway [30,31]. For example, *Chlamydia trachomatis* infection induces the mRNA expression of STATs (STAT1 and STA2) and promotes STAT1 activation in HeLa 229 cells [31]. In vivo experiments on *Drosophila*, *Anopheles gambiae*, and other invertebrates, the JAK-STAT signaling pathway appeared to be a key regulator of the innate immune response against bacterial infection [30,32]. For the first time, this study discusses the effects of recombinant ApSOCS-2 protein administration, STAT, and *ApSOCS-2* knockdown on mRNA expression AMPs in *A. pernyi*. The recombinant ApSOCS-2 protein administration strongly down-regulated all the tested AMPs under bacterial infection (*M. luteus*). In the *ApSOCS-2* knockdown *A. pernyi* larvae, AMPs expression was significantly induced, suggesting that in *A. pernyi* larvae, the *ApSOCS-2* gene is required to modulate the production of AMPs and mount a host protective immune response against the bacterial infection. In addition, following RNA interference of *ApSTAT*, the mRNA expression of *ApSOCS-2* was significantly reduced, indicating that the JAK-STAT pathway modulated *ApSOCS-2* expression in *A. pernyi*. In vertebrates, SOCS proteins participate in a negative feedback loop of the JAK-STAT pathway because they are transcriptionally activated via JAK-STAT signaling [33]. Taken together, STAT appears to regulate the expression of AMPs and *ApSOCS-2* transcript. Therefore, we concluded that SOCS-2 might negatively modulate the JAK-STAT signaling cascade via inhibiting STAT activation in *A. pernyi* larval innate immune response. We also demonstrated that the JAK-STAT pathway directs the production of AMPs against bacterial (*M. luteus*) infection, which is also known in other invertebrates, like *Marsupenaeus japonicus*, *B. mori* [22,34].

Our transcriptomic analysis revealed that the genes related to JAK-STAT and Toll pathways were strongly upregulated after *ApSOCS-2* knockdown. Besides, the JAK-STAT pathway has been shown to play an active role in the host defense, approximately similar to that of the Toll pathway [11,35]. It seems that both the JAK-STAT and Toll pathways are activated simultaneously to attenuate the invaded microbial pathogen. These results are in agreement with a previous study, which showed that different immune pathways, including JAK-STAT, Toll, and IMD pathways, are induced in response to Wolbachia infection. Another study suggested that these immune pathways seem to have crosstalk for the defense of host animals [36]. Further studies need to clarify how the JAK-STAT pathway cooperates with the other immune pathways to suppress microbial infection. Many cell surface receptors were also upregulated, such as integrins, which play a crucial biological role in the elimination of pathogens by regulating encapsulation, pathogen adherence, and phagocytosis [37,38,39]. Heat-shock proteins, conserved across invertebrates, also showed upregulation, which are involved in stress-related and immune responses [40,41]. The antioxidant genes, including Glutathione-S-transferase, and cytochrome P450 members, are essential in protecting against the damage of reactive oxygen species produced to kill bacteria [42,43,44]. Collectively, knockdown of *ApSOCS-2* influences the expression of a large variety of genes, which may directly or indirectly contribute to the elimination of bacterial infection. However, future studies will validate how SOCS-2 influences other immune signaling pathways besides the JAK-STAT pathway and other genes related to immune responses.

## 4. Materials and Methods

### 4.1. Experimental Animals

Chinese oak silkworm (*Antheraea pernyi*) larvae were reared in the State Key Laboratory of Silkworm Genome Biology, Southwest University, China. The larvae were fed on fresh oak leaves and maintained at room temperature (26 ± 1 °C), 10:14 h light: dark photoperiod, and 70% relative humidity. Only healthy 5th instar larvae of *A. pernyi* were taken in all the experiments.

### 4.2. Identification and Bioinformatics Analysis of ApSOCS-2

The full-length *ApSOCS-2* sequence was identified using the *A. pernyi* transcriptomic data. Subsequently, the *ApSOCS-2* open reading frame (ORF) sequence was mapped in the genomic sequence using the NCBI ORF finder (https://www.ncbi.nlm.nih.gov/orffinder/ accessed on 16 February 2019). The conserved domains were predicted using the Smart 4.0 program [45], and NCBI conserved domain finder. The multiple sequence alignment, percentage identity of ApSOCS-2 amino acid, and orthologous sequences from different species were executed using the ClustalX2 program. The phylogenetic evolutionary tree was constructed using the maximum likelihood method by the MEGA 6.0 program. The NCBI database was utilized to retrieve homologous protein sequences from different invertebrate and vertebrate species, and the GenBank accession numbers of the protein sequences used are given in Table S2.

### 4.3. Recombinant Protein Expression and Purification

To express the recombinant ApSOCS-2 protein in the prokaryotic expression system, a pair of specific primers was designed to amplify the ORF of *ApSOCS-2* (Table S1). The obtained ORF sequence was cloned into pMD-19 T and digested with Bam HI and Xho I, then ligated into the Pet 30a vector. The insert (i.e., Pet 30a-*ApSOCS-2*), was then confirmed by DNA sequencing and transformed into *E. coli* (BL21 DE3) (Novagen, USA) for protein expression. After short-term culturing and an IPTG induction at a final concentration of 0.8 mM, the cells were harvested by centrifugation at 8000× *g* for 5 min. The cell pellets were suspended in a binding buffer containing 20 mM Tris–HCl, 500 mM NaCl, 5 mM imidazole, pH 7.9, and disrupted by sonication on ice. After centrifugation at 12,000× *g* for 20 min at 4 °C, the recombinant ApSOCS-2 protein was purified using affinity chromatography. To analyze the recombinant ApSOCS-2 proteins, we used 15%-sodium dodecyl sulfate-polyacrylamide gel electrophoresis (SDS-PAGE).

### 4.4. Tissue Distribution Analysis and Immune Challenge Studies

To analyze tissue distributions of *ApSOCS-2* at the mRNA level, quantitative real-time PCR (qRT-PCR) was conducted [35,39,46]. In brief, for tissue distribution, total RNA was extracted from the 5th instar at 3rd day larval tissues, such as fat body, hemocytes, integument, gut, and Malpighian tubules. Notably, hemocytes were sampled as pellets after centrifugation of the hemolymph at 6000× *g* for 6 min. For gene tissue distribution analysis, nine Chinese oak silkworm larvae were used (three larvae were pooled together as one group). Tissues were sampled from each of the groups to make three samples from each tissue.

For immune challenge studies, healthy *A. pernyi* larvae were micro-injected intra-abdominally with *E. coli*, *M. leutus*, *B. bassiana*, or ApNPV, respectively. PBS was used as a control. The injection sites were sealed with Vaseline immediately following injection. Three larvae were collected at different time points (1.5, 3, 6, 12, 24, and 48 h) post-injection, and total RNA was isolated at each time point by the Trizol Reagent (Takara, Dalian, China), and the first-strand cDNA was synthesized using TransScript Synthesis SuperMix (TransGen, Beijing, China) according to the manufacturer’s instructions. Actin-1 (GenBank: KC242321.1) and 18s ribosomal RNA (Accession number: DQ347469) genes were used as an internal control for normalizing mRNA expression. All experiments were executed in triplicate and are shown as mean ± standard error of three biological replications.

### 4.5. ApSOCS-2 Gene Silencing

For *ApSOCS-2* RNA interference, a fragment of *ApSOCS-2* was amplified by specific primers comprising the T7 promoter sequences at the 5′-ends. As a negative control, a fragment of the green fluorescent protein (GFP) gene was used as a template for the synthesis of GFP RNA. The polymerase chain reaction (PCR) was performed at an initial denaturation of 95 °C for 5 min, followed by 35 cycles of denaturation at 95 °C for 10 s, annealing at 56 °C for 30 s, and extension at 72 °C for 50 s. The PCR product was resolved on the agarose Gel, and the purified product was then used to synthesize *ApSOCS-2* double-strand RNA using T7 RiboMAX™ Express RNAi System (Promega) following the manufacturer’s instructions. A total of 5 µL (2 µg/1 µL) synthesized *ApSOCS-2* dsRNA was injected intra-abdominally into the 5th instar larvae of *A. pernyi*. The GFP dsRNA injected and untreated larvae were used as a control. The experiment was performed in triplicate to ensure the suppression of *ApSOCS-2* transcripts.

### 4.6. Effect of ApSOCS-2 Depletion or Recombinant Protein Administration on AMP Gene Expression

To determine the biological role of *ApSOCS-2* in modulating the mRNA expression of AMPs, including cecropin, moricin, lebocin, and gloverin, microbial pathogens were injected into *ApSOCS-2-*knockdown *A. pernyi* larvae. A GFP dsRNA injected and untreated insects were used as control. Fat body samples were collected and processed for total RNA extraction, cDNA synthesis, and qPCR analysis were performed using AMPs primers. Furthermore, the mRNA profile of these AMPs was also quantified following the administration of recombinant ApSOCS-2/*M. luteus*, while the BSA/*M. luteus, M. luteus*, or the untreated *A. pernyi* larvae were used as controls. After 3, and 6 h of microbial administration, fat body samples were collected and used to synthesize cDNA for qRT-PCR analysis.

### 4.7. Antibacterial Activity of Plasma

To understand the effect of an *ApSOCS-2* knockdown on the antibacterial activity of larval plasma, hemolymph samples were collected from *the A. pernyi* 24 h and 48 h after the *dsApSOCS-2* (experimental group), or *dsGFP* (control group) injections, and likewise from the untreated sample serving as the negative control. The assay followed that of Kausar et al. [16], but with a slight modification. The collected hemolymph was initially centrifuged at 800 rpm for 10 min to separate it from the hemocytes and then centrifuged at 12,000 rpm for 20 min to obtain a clear supernatant. Equal volumes (10 μL) of the supernatant and bacterial suspension (*M. luteus*) were mixed together and stored at 25 °C for 1 h. These mixtures were then diluted, plated onto LB agar plates, and cultured at 37 °C for 30 h, and the ensuing colony counts were recorded. These results were expressed as the mean ± SD derived from three independent repeats.

### 4.8. DNA Isolation and qPCR

To analyze bacterial replication in the infected *A. pernyi* larvae, total genomic DNA was isolated from the larval hemolymph samples of treated (bacterial-injected and RNAi treated) and control larvae. Genomic DNA concentrations were determined by a spectrophotometer (Epoch, BioTek, Winooski, VT, USA), and 500 ng total genomic DNA was used for each qPCR reaction using SYBR Green as described above. Specific primers to 16 s rRNA of the bacteria were used in qPCR [47], and the *A. pernyi* actin-1 and 18 s rRNA gene was used as an internal control for normalization (Table S1). For each experiment, three biological replicates were replicated three times.

### 4.9. Effect of ApStat Knockdown on ApSOCS-2 Expression

To determine expression patterns of *ApSOCS-2* following the suppression of *ApStat*, *A. pernyi* larvae were treated with *dsApStat* and then challenged with *M. luteus* after 24 h of RNAi treatment. Fat body and hemocyte samples were collected after different time points (6 and 12 h) following the bacterial treatment. The samples without any treatment or *dsGFP* treated were used as controls. The samples were processed RNA extraction and then cDNA synthesis, and the level of *ApSOCS-2* was analyzed by performing qRT-PCR as described before.

### 4.10. Transcriptome Analysis

The fat bodies of *A. pernyi* larvae on the 3rd day of the 5th instar were sampled following injection of *dsApSOCS-2* and *M. luteus* or *dsGFP* and *M. luteus* challenge. Three independent biological replicates were executed for each treatment, and each collected sample contained fat bodies from three larvae. Total RNAs were isolated, and cDNA libraries were constructed by Illumina TruSeq RNA preparation kits according to manufacturer protocol and then sequenced by the HiSeq™ 2000 Sequencing System (Illumina, San Diego, CA, USA). Raw data (raw reads) in fastq format was firstly processed through in-house perl scripts. The clean reads were generated by removing reads containing adapters, poly-N, and low-quality reads using Cutadapt. At the same time, Q20, Q30, and GC content of the clean data were calculated. All the downstream analyses were based on clean data of high quality. The transcriptome was de novo assembled with Trinity 2.5.1 [48]. Trinity is a full-length transcriptome assembly tool from RNA-Seq data without a reference genome (https://github.com/trinityrnaseq/trinityrnaseq/wiki Accessed on 16 February 2019). The gene transcript levels were measured as fragments per kilobase per million mapped reads (FPKM) using RSEM v1.2.19. Differential expression analysis of two groups was performed using DESeq2 v1.6.3 (http://www.bioconductor.org/packages/release/bioc/html/DESeq.html Accessed on 16 February 2019). The differentially expressed unigene were detected with log2 (fold change) > 1 or log2 (fold change) < −1 and the value of false discovery rate was < 0.01. The functional annotation of sequence data was determined by Blast2GO v2.5. The KEGG analysis for differentially expressed genes was performed based on the database of the Kyoto Encyclopedia of Genes and Genomes (http://www.genome.jp/kegg/ Accessed on 16 February 2019) using KOBAS 2.0. The heat map was created using an online tool (https://software.broadinstitute.org/morpheus/ Accessed on 16 February 2019).

### 4.11. Statistical Analysis

For qRT-PCR-based expression analysis of *ApSOCS-2* transcripts, the 2^−ΔΔCt^ method was used. Data were subjected to one-way analysis of variance (ANOVA) and Tukey’s multiple range tests to evaluate differences between groups (* *p* < 0.05, ** *p* < 0.01, *** *p* < 0.001).

## 5. Conclusions

In summary, we identified the SOCS-2 gene from *A. pernyi*, which was expressed in all the tested tissues. Its mRNA expression was induced after viral, fungal, and bacterial pathogens in fat bodies and hemocytes. The recombinant ApSOCS-2 protein strongly reduced the expression of AMPs. In contrast, loss-of-function studies using RNAi exhibited a significant increase in AMPs production. Further, the knockdown of *ApStat* strongly impaired the mRNA expression levels of *ApSOCS-2*, suggesting that the target gene is involved in immune defense against bacteria by negatively regulating the production of AMPs via the JAK-STAT signaling pathway.

## Data Availability

The data presented in this study are available in the article and Supplementary Material.

## Supplementary Materials

The following supporting information can be downloaded at https://www.mdpi.com/article/10.3390/ijms231810389/s1.
