# Supplementary material for: Antheraea pernyi Suppressor of Cytokine Signaling 2 Negatively Modulates the JAK/STAT Pathway to Attenuate Microbial Infection
Source: Int J Mol Sci. 2022 Sep 8;23(18):10389. doi: 10.3390/ijms231810389 (PMC9499667; doi:10.3390/ijms231810389)
Supplement: Supplementary file 1 [file ijms-23-10389-s001.zip › Figures and Tables (Supplementary materials).pdf]

```
   1 GTCGAGTATAGTAGAAGGTATATGTATAACATTACGCAATCTGTTTCACCTACGGCTCGG
  61 TGCGGACGGATAACTTTTCCATTAACGCTGTGCACTGAATTGAACATACACTCATATGGC
 121 GCTAACCAACAAAAAATAGACCAAGTGAATGAACTTTCTAACGTTTATTAGACGTCTGAA
 181 AGCGACGGGGCCCTGCCTCCAACATTGATTGATGGATAGATAGATACCAAGAGTTACCTA
 241 ATTTAACATTGTGCGCCGGTCATCGGGGTCAACAGTCCACCGTAAAGTTAGTTAAGAGTC
 301 ACGAACCGCCATGACAGAACCGGGCCGTCGTGTTCGCCAGCCTTGGACGGAAGAACATAA
 361 GTTTTATAAATTAGTGACAATGCCTTGAGTTATTTCGTGGAAGCGACAAGGTTGGCCGTT
 421 CTGGGAAGTGGCGGCTCGTCGGCAAGTACGACTTGCTCGTGCTCGGCGTCCCCGCACTCC
 481 CCGCTGCCGCGCCCGCACCCCGCACGCGTGTTTATTTCACGTCCGAACACACCGCGCGCC
 541 AGCTATACTCCACGCCGAGTCGTTCAGTCTGAATTCGTGCACGGAAGAGAGAGCCGTCAT
 601 CGCCGCGACGCAACGATCACGAACTTTCACCTCCGTGATATTATCACCTGTGATAAACAC
 661 CGATGCCGGGTGAAACTGAATGTGCAGTCCGTACTTAGAAAGCGTTTCTGTCGCTGTGAA
 721 CGGACTGCCGACCGTCGGTTTCTGCAAATTAATTTCTCCAGTGTAGTTGTCGGTTTCTTC
 781 GAAACTGCTTGTAAAGAGTTTTACTGAGAAACGAAACGGCCGTAAAGTGGATTTGAGGAA
 841 ATATAAGTAATTATAAGTGGGCTTTTGTGTTTCCGTGTCTGTTGTTAACAAAGACGTTTA
 901 CATTGGATCCCAATCGAACATTCACACCTCCAATGGTGGTTAAACTACCAGATGCATGCG
   1                                    M  V  V  K  L  P  D  A  C
 961 TGGAAGTGGGTGTTCCACTGAATAGTTGGTCAACAGGTGTGGCATGTTGTCCTAACTGCA
  10 V  E  V  G  V  P  L  N  S  W  S  T  G  V  A  C  C  P  N  C
1021 GGCACGAGCTGAGGGTTTCCCTCGCGCCGGCAGGTGGGAAATGCCACGCTTTACCGTCGC
  30 R  H  E  L  R  V  S  L  A  P  A  G  G  K  C  H  A  L  P  S
1081 CACACACGCCGCCACCGTTCACGATCCAGCCGACTTACTTGCCTTATTCACCGCTGTACG
  50 P  H  T  P  P  P  F  T  I  Q  P  T  Y  L  P  Y  S  P  L  Y
1141 CTACACCTTCGTCTCCCATTATACCTACACCGTACAACGATGAACTCAGACGACTCGCAG
  70 A  T  P  S  S  P  I  I  P  T  P  Y  N  D  E  L  R  R  L  A
1201 ACACTCTCAGAGCGTTGCGTTTGTCCGGGTGGTACTACGGGAATTTGGATTGGCAGGGTG
  90 D  T  L  R  A  L  R  L  S  G  W  Y  Y  G  N  L  D  W  Q  G
1261 CAAGAACATTGTTGAAAGACGCGAGTGTCGGAGCGTTTGTGATCCGAGACTCCGGAGACA
 110 A  R  T  L  L  K  D  A  S  V  G  A  F  V  I  R  D  S  G  D
1321 GGAACTTTATATTCTCATTGTCCGTGCAAACGGAAAGGGGACCCACATCAGTGCGATTGC
 130 R  N  F  I  F  S  L  S  V  Q  T  E  R  G  P  T  S  V  R  L
1381 ATTACGAGCAAGGCTTTTTCAGATTAGACTGTGACCTACCGCTCGCAAGGTACATGCCAA
 150 H  Y  E  Q  G  F  F  R  L  D  C  D  L  P  L  A  R  Y  M  P
1441 GATTCAGATGCGTCGTTGAACTGGTCCAGCACTACACGAGAGTAGGCGACAGAGGCGCGG
 170 R  F  R  C  V  V  E  L  V  Q  H  Y  T  R  V  G  D  R  G  A
1501 GAGGCACCGTGTGGGTAGACAGAGAGGGTTGCCCGCACTCTCCAGTACTCCTCAAAGTAC
 190 G  G  T  V  W  V  D  R  E  G  C  P  H  S  P  V  L  L  K  V
1561 CACTCCGGCAAAGTCCACCTTCCCTCCTCCACGCAGCGAGGCTCGCCGTCCACAAAGCCC
 210 P  L  R  Q  S  P  P  S  L  L  H  A  A  R  L  A  V  H  K  A
1621 TGGACTCGAACCCGCTGACACCGAAGCTCTGGTGTGCCCCCAAACACAGGCTCCTACCTC
 230 L  D  S  N  P  L  T  P  K  L  W  C  A  P  K  H  R  L  L  P
1681 TTCCATCAACGCTTATAGACTATCTCGGTGAATACCCGTACTCGATCTAACACCAAGATC
 250 L  P  S  T  L  I  D  Y  L  G  E  Y  P  Y  S  I  *
1741 TGATATAGTTGATTAATCCGAGTGATGAGTGAAGATATTAGTCGATTTAGTTAACTAACT
1801 CGATAATCGATCACACTAACAGTTAATCATCGCCTCAGCATTATCATAATATATAATTAT
1861 TTTGTACAAAATGTATAATAATTAATGGAAATACTTAGCACAGGTTGCTAATTAGGGCCG
1921 GATCCTATAAATAACGTTTAACAATGTATATTTATGTTTAAATTAACTGTATTAAATAAT
1981 AAGTATTGAAAATGGGACTCAACTACTATGGTATTTAGTGAAAAATATTTACGCAGTCTA
2041 TCGTTTAAGCCGTTCCGTGTTTGTATATAGGGATTTTGTTTGAAAGTTGAAGACTGATTT
2101 CCTAGAAGTCTGTAATTATGTTTTTGTGATGTGTGAATTTCTTTATAATTTAACATTCAT
2161 ATATATTCTGAGTTTTTTTTTTAAATATTGACTGTAAGACGGAAATTTACTCAGTGTATG
2221 ATTTCATTATTATATATTTTAATTTTCTTTTACGGTAATTATCATGTATCGATATTAATT
2281 CCTGTTCTAGTTTAATCGTCTCGAAGGACCATTATGAAAGTGAAAATTCGTGTATAAATA
2341 AATTTCATATATAATTTTAT(n)
```

Figure S1: Nucleotide and deduced amino acid sequences of *ApSOCS-2*. The nucleotides are numbered from the first base of the cDNA (ORF starts from 933), representing the start of the gene. The SH2 domain extending from "amino acid" position 98 to 184 is exhibited in a brown shade. The SOCS-box extending from position 216 to 256 is shown in a light green shade. The conserved domains are shaded. The poly (A) signals (AATAAA) were underlined with red colour. The 3′-UTR instability motifs (ATTTA) were boxed with blue colour.

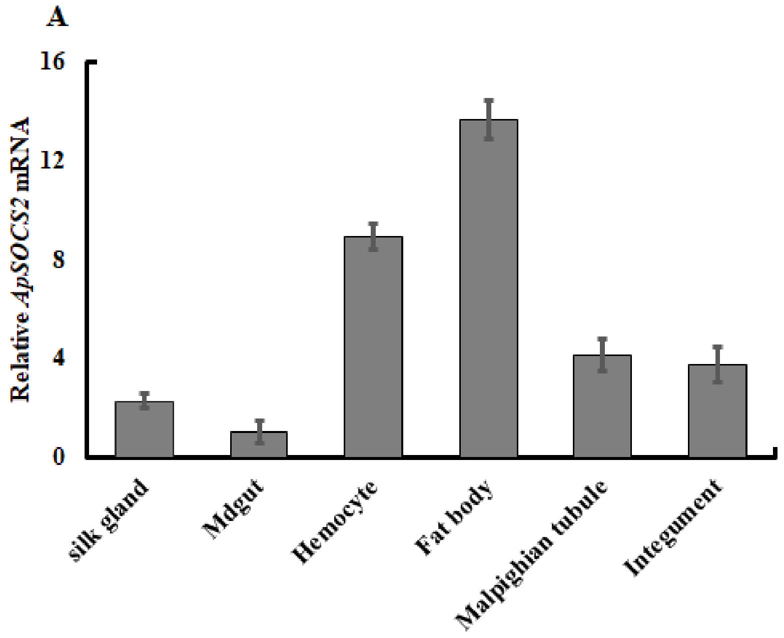

Figure S2. Expression profile of *ApSOCS-2* in various tissues of *A. pernyi* normalized to actin-1. The mRNA expression profile of *ApSOCS-2* in various tissues of the *A. pernyi* was determined using qRT-PCR. The values are represented as mean ± S.E.

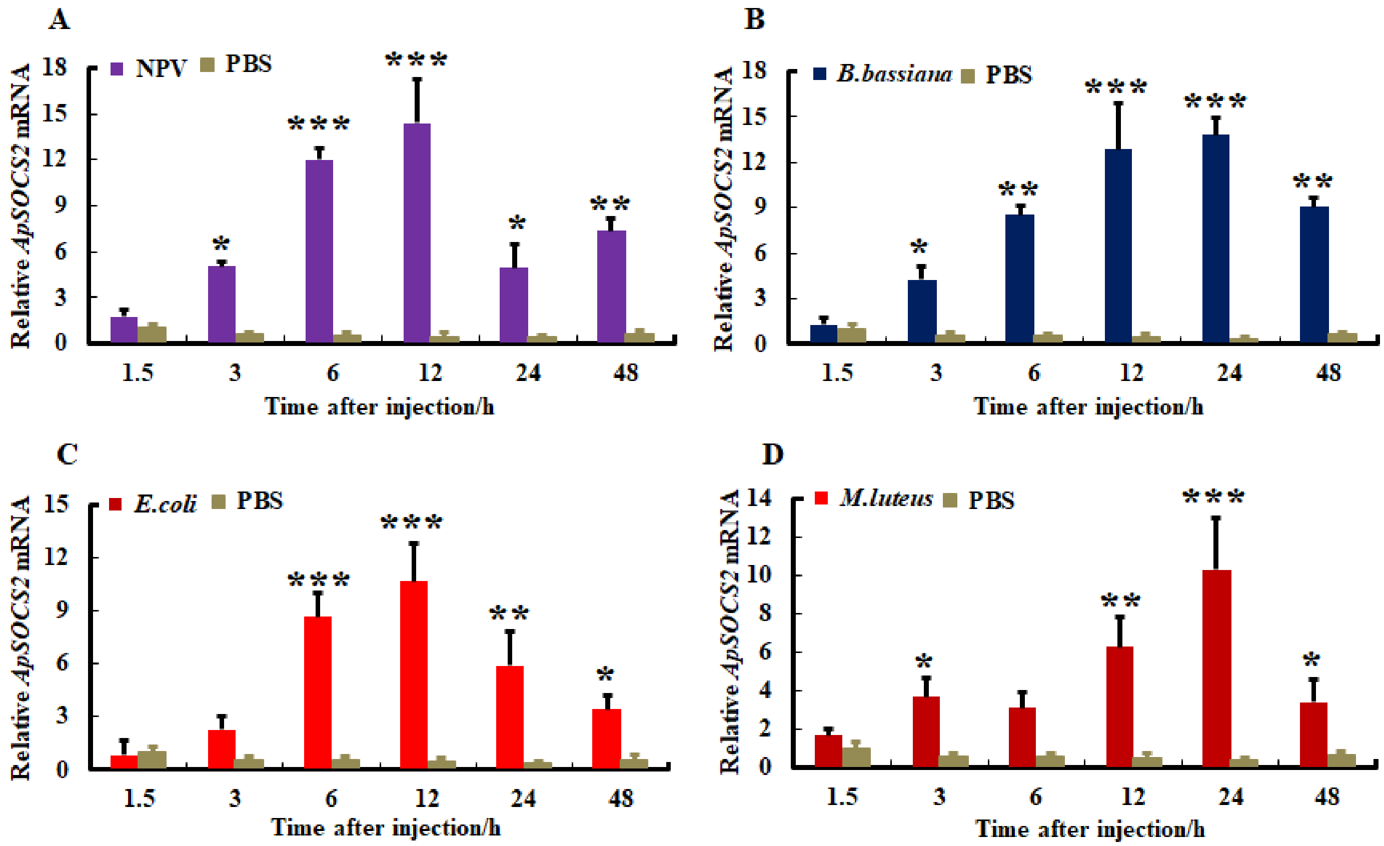

Figure S3. The mRNA expression levels of *ApSOCS-2* in fat bodies following microbial challenge normalized to actin-1. Following injection of A: ApNPV, B: *B. bassiana*, C: *E. coli*, D: *M. luteus*, the mRNA expression levels of *ApSOCS-2* were analyzed using qRT-PCR. Data of triplicate experiments (n = 3) are represented with mean ± S.E. Asterisks indicate significant differences (* $p < 0.05$, ** $p < 0.01$, *** $p < 0.001$).

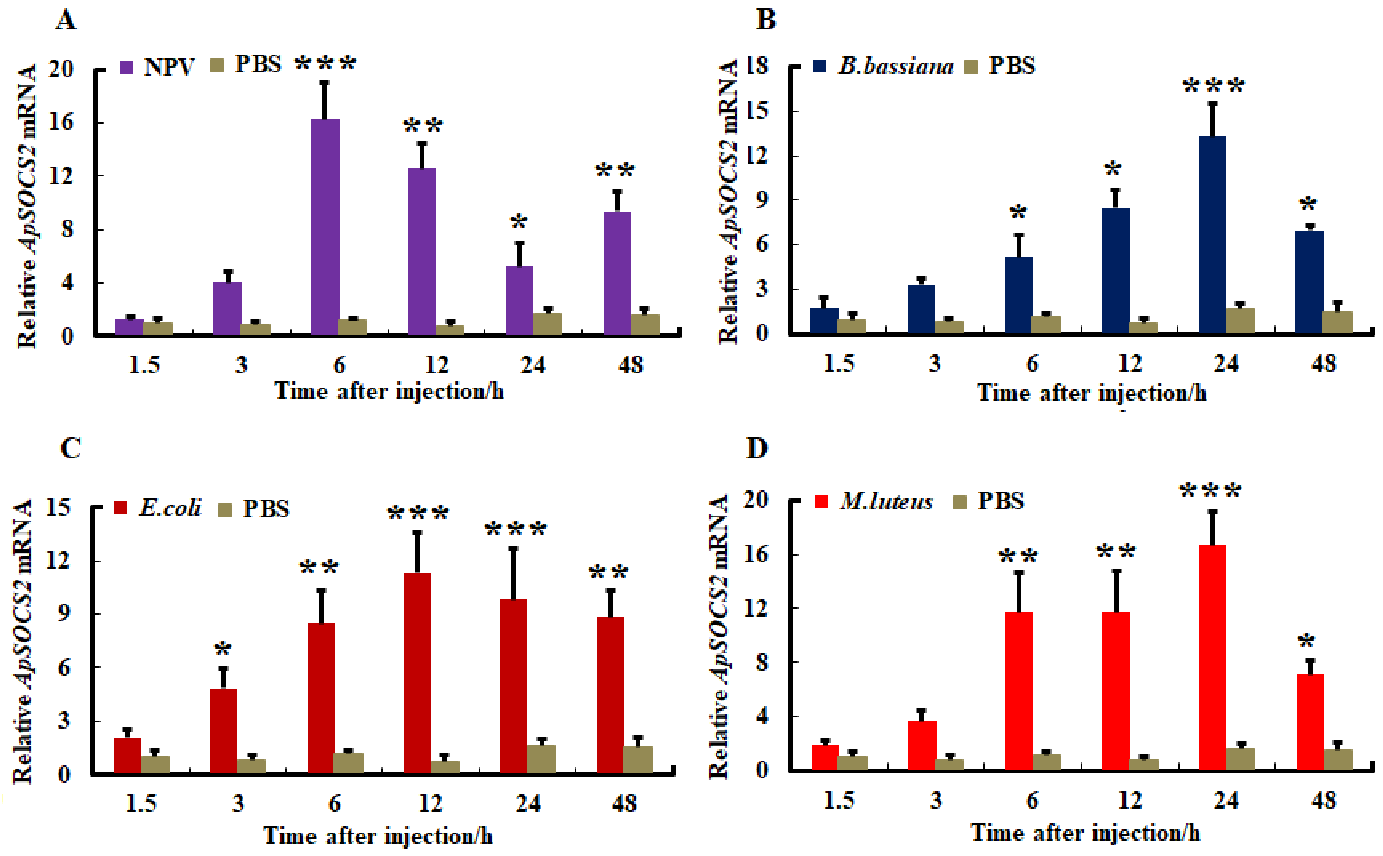

Figure S4. The mRNA expression levels of *ApSOCS-2* in hemocytes following microbial challenge normalized to actin-1. Following injection of A: ApNPV, B: *B. bassiana*, C: *E. coli*, D: *M. luteus*, the mRNA expression levels of *ApSOCS-2* were analyzed using qRT-PCR. Data of triplicate experiments (n = 3) are represented with mean ± S.E. Asterisks indicate significant differences (* $p < 0.05$, ** $p < 0.01$, *** $p < 0.001$).

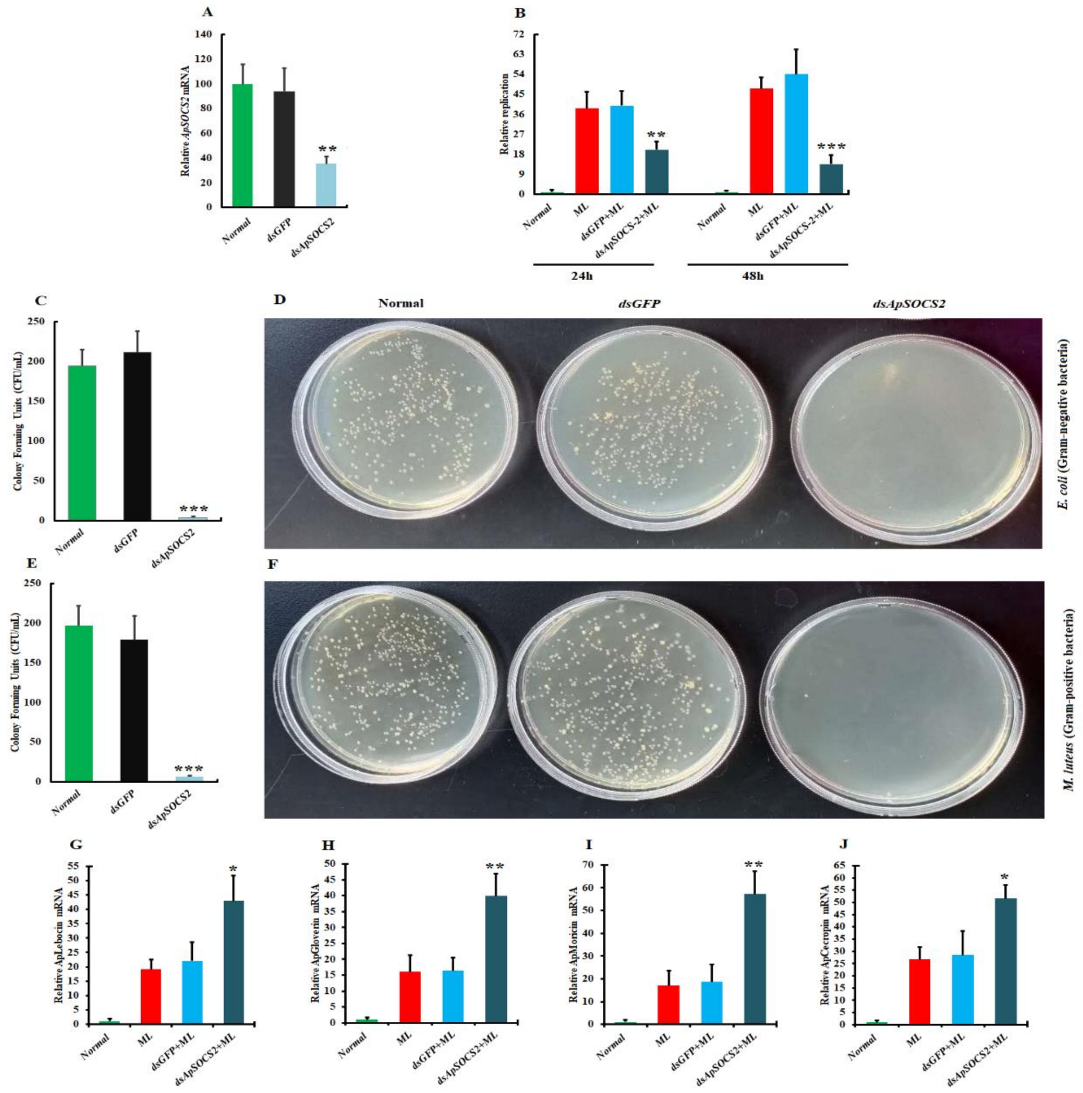

Figure S5. Loss-of-function analysis of *ApSOCS-2* on bacterial survival and antimicrobial peptides production normalized to actin-1. A: *dsApSOCS-2* injection strongly reduced the production of *ApSOCS-2* in hemocytes; B: qPCR replication rate analysis exhibited that treatment of *A. pernyi* larvae with *dsApSOCS-2* decreased the replication rate of *M. luteus*. The plasma antibacterial activity was

increased in the *ApSOCS-2* depletion (C-F). Plasma was sampled from *A. pernyi* treated with *ApSOCS-2* dsRNA or GFP dsRNA. Equal volumes (10 μl) of plasma and bacterial suspension were incubated at 25 °C for 1 h. Bacteria (*M. luteus*) was injected into *dsApSOCS-2* treated *A. pernyi* larvae, and mRNA expression levels of (G) lebocin, (H) gloverin, (I) moricin, and (G) cecropin were measured by qRT-PCR. Normal: untreated sample; ML: *M. luteus* -treated; *dsGFP*+ML: treated with both *dsGFP* and *M. luteus*; and *dsApSOCS-2*+ML: treated with both *dsApSOCS-2* and *M. luteus*. Asterisks indicate significant differences (* $p < 0.05$, ** $p < 0.01$, *** $p < 0.001$).

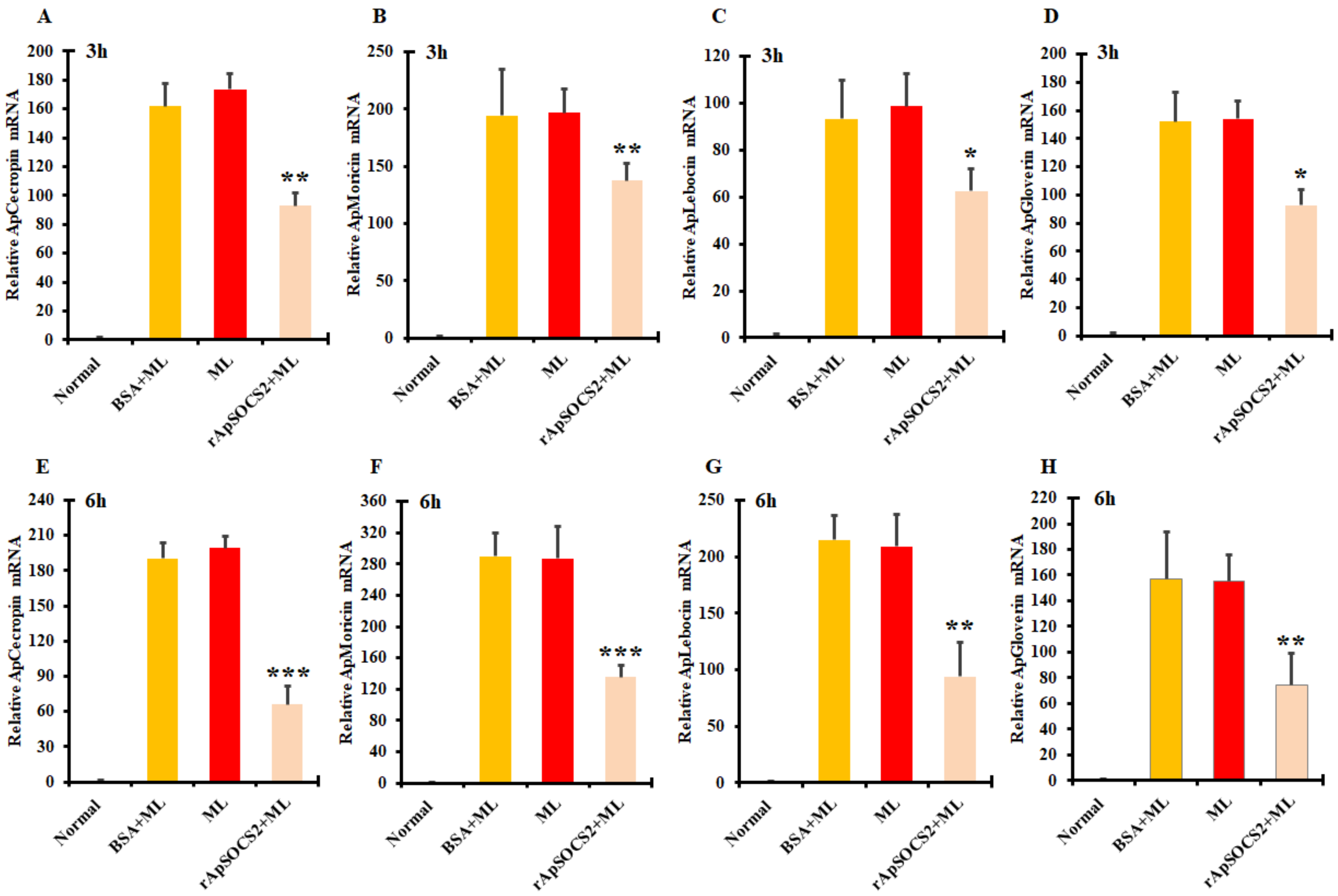

A
3h
Relative ApCecropin mRNA
**
B
3h
Relative ApMoricin mRNA
**
C
3h
Relative ApLebocin mRNA
*
D
3h
Relative ApGloverin mRNA
*
E
6h
Relative ApCecropin mRNA
***
F
6h
Relative ApMoricin mRNA
***
G
6h
Relative ApLebocin mRNA
**
H
6h
Relative ApGloverin mRNA
**
Normal
BSA+ML
ML
rApSOCS2+ML

Figure S6. Relative mRNA expression levels of the pertinent AMPs, namely cecropin, moricin, lebocin, and gloverin (A–D) at 3 h after the recombinant ApSOCS-2 protein injection and (E–H) at 6 h after the recombinant ApSOCS-2 protein injection normalized to actin-1. Normal: untreated; BSA+ML: treated with bovine serum albumin and *M. luteus*; ML: *M. luteus*-treated; and recombinant ApSOCS-2+ML: treated with both recombinant ApSOCS-2 protein and *M. luteus*. Asterisks indicate significant differences (* $p < 0.05$, ** $p < 0.01$, *** $p < 0.001$).

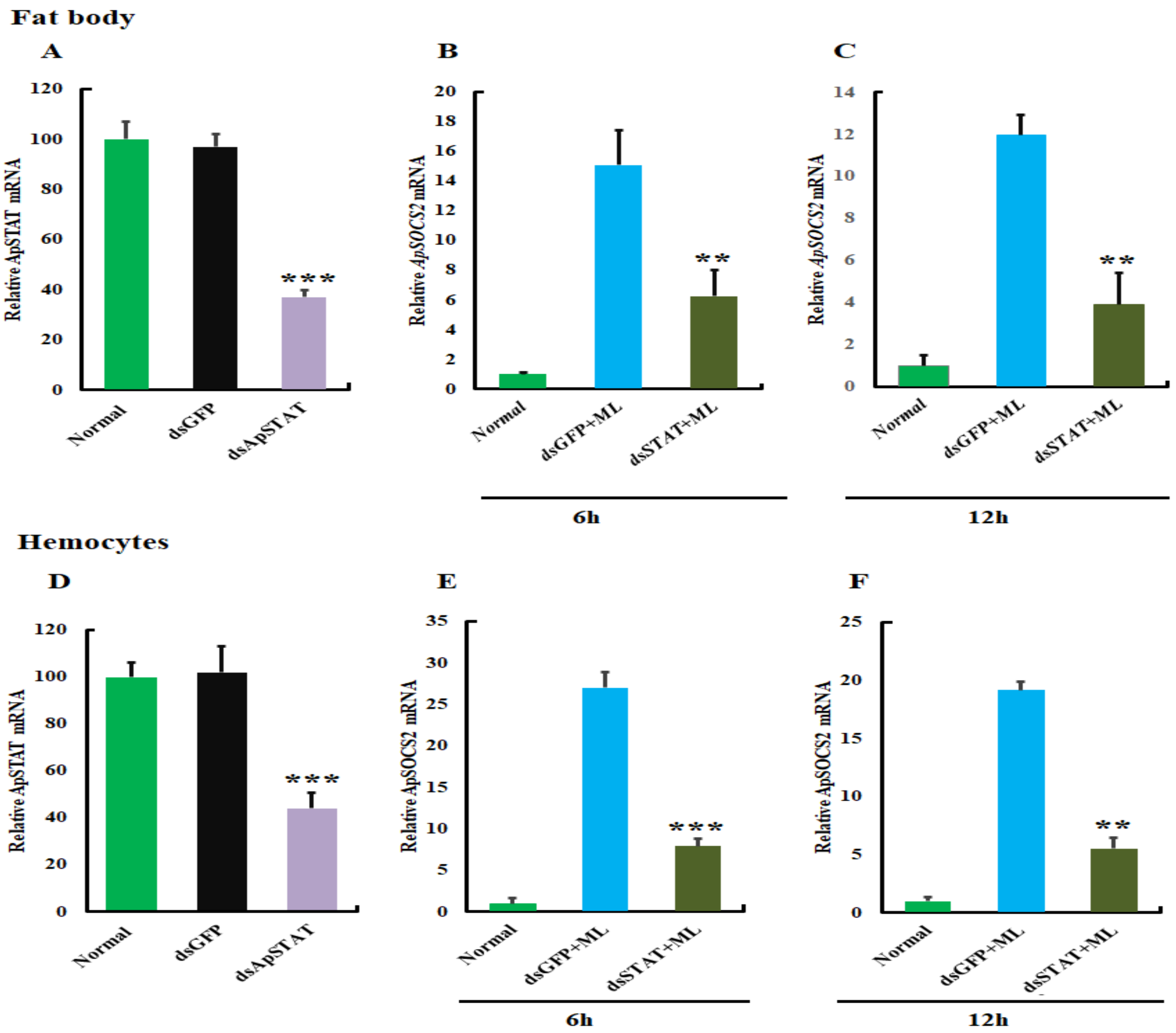

Fat body
A
B
C
Relative ApSTAT mRNA
Relative ApSOCS2 mRNA
Normal
dsGFP
dsApSTAT
dsGFP+ML
dsSTAT+ML
***
**
6h
12h
Hemocytes
D
E
F
Relative ApSTAT mRNA
Relative ApSOCS2 mRNA
Normal
dsGFP
dsApSTAT
dsGFP+ML
dsSTAT+ML
***
**
6h
12h

Figure S7. *ApStat* regulates *ApSOCS-2* expression in silkworm challenged with *M. luteus* at 6 and 12 h normalized to actin-1. The efficiency of *ApStat*-RNAi in fat bodies (A) and hemocytes (D). (C-D) After *dsApStat* injection, the *ApSOCS-2* expression in fat bodies of silkworm challenged with *M. luteus* at 6 (B) and 12 h (C) was detected by qRT-PCR in fat bodies. Following *dsApStat* injection, the *ApSOCS-2* expression in hemocytes of silkworm challenged with *M. luteus* at 6 (E) and 12 h (F) was detected by qRT-PCR. The GFP-RNAi was used as the control. Asterisks indicate significant differences (* $p < 0.05$, ** $p < 0.01$, *** $p < 0.001$, *** $p < 0.001$).

Table S1. Primers used in this study.

Primer Name	Fwd./ Rev.	Purpose	Sequence (5′-3′)	Reference
ApSOCS-2	Fwd.	Amplification of ORF	AGACGTTTACATTGGATCCC	This study
	Rev.		TACGGGTATTCACCGAGATAG	
ApSOCS-2	Fwd.	Recombinant expression	CGGGATCCGTGGTTAAACTACCAGA	This study
	Rev.		CCGCTCGAGTCTCGTGTAGTGCTGGA	
ApSOCS-2	Fwd.	RNAi	TAATACGACTCACTATAGGCCGACTTACTTGCCTTA	This study
	Rev.		TAATACGACTCACTATAGGGAGTGGTACTTTGAGGAG	
GFP	Fwd.		TAATACGACTCACTATAGGATGGTGAGCAAGGGCGAGGA	[31]
	Rev.		TAATACGACTCACTATAGGTTACTTGTACAGCTCGTCCA	
ApStat	Fwd.		TAATACGACTCACTATAGGTTGTTTGGCGCCATGGATTG	This study
	Rev.		TAATACGACTCACTATAGGCGTCTCCTCGATCTGACTGC	
ApSOCS-2	Fwd.	qPCR	CACGATCCAGCCGACTTACT	This study
	Rev.		GACACTCGCGTCTTTCAACA	
ApStat	Fwd.		AAGCTGACGTTGCTCTTCCA	This study
	Rev.		GACCCCACGTCACCTTATCG	
Ap18s rRNA	Fwd.		CGATCCGCCGACGTTACTACA	[18]
	Rev.		GTCCGGGCCTGGTGAGATTT	
ApActin-1	Fwd.		TCGCGATCTCACAGACTACC	This study
	Rev.		TCGTAGGACTTCTCGAGGGA	
ApCecropin	Fwd.		CGTCCTGGCTTTGACAACAG	This study
	Rev.		TCTCCTAAAACTGCTACCGC	
ApMoricin	Fwd.		TCTTATTTTGTCGATCCTTGCG	This study
	Rev.		CAGCACTGATCACACGGAAC	
ApLebocin	Fwd.		ATGATGATGCTCCGGGTGAA	This study
	Rev.		CCTCCAGAAAGACCCATCGT	
ApGloverin	Fwd.		CCCGTCGTCATTTTGTCCAA	This study
	Rev.		GGCAAGCGTCTACTCACAAG	

16s rRNA	Fwd.		AGAGTTTGATCMTGGCTCAG	[48]
	Rev.		TACGGYTACCTTGTTACGACTT	

Table S2. List of species, genes and their accession numbers used in this study.

Sr. no.	Gene	Species	Accession no.
	**CIS**		
1		*Mus musculus*	NP_034025
2		*Homo sapiens*	AAH31590
3		*Danio rerio*	NP_001070085
	**SOCS-1**		
4		*Danio rerio*	NP_001003467
5		*Homo sapiens*	NP_003736
6		*Mus musculus*	NP_034026
7		*Rattus norvegicus*	NP_665886
	**SOCS-2**		
8		*Papilio xuthus*	KPJ02665
9		*Papilio machon*	KPJ08265
10		*Danaus plexippus*	EHJ63976
11		*Antheraea pernyi*	Present study
12		*Tribolium castaneum*	EFA08217
13		*Eriocheir sinensis*	ACU42699
14		*Crassostrea gigas*	EKC24772
15		*Rattus norvegicus*	NP_478115
16		*Homo sapiens*	NP_001257400
	**SOCS-3**		
17		*Oncorhynchus mykiss*	NP_001139640
18		*Homo sapiens*	NP_003946
19		*Mus musculus*	NP_031733
20		*Rattus norvegicus*	NP_446017
	**SOCS-4**		

21		*Drosophila melanogaster*	NP_523593
22		*Homo sapiens*	NP_543143
23		*Mus musculus*	NP_543119
	**SOCS-5**		
24		*Danio rerio*	NP_001107269
25		*Homo sapiens*	NP_659198
26		*Mus musculus*	NP_062628
27		*Rattus norvegicus*	NP_001102744
	**SOCS-6**		
28		*Bombyx mori*	NP_001185652
29		*Drosophila melanogaster*	NP_523659
30		*Rattus norvegicus*	NP_001258078
31		*Oncorhynchus mykiss*	CAP17278
32		*Homo sapiens*	NP_004223
	**SOCS-7**		
33		*Drosophila melanogaster*	NP_523390
34		*Homo sapiens*	NP_055413
35		*Oncorhynchus mykiss*	NP_001182101
36		*Danio rerio*	NP_001274006
37		*Mus musculus*	NP_619598

Table S3. Statistics of the transcriptome sequence data.

	**Clean Bases**	**Clean Reads**	**Mapped Reads**	**Mapped Ratio (%)**	**Q20%**	**Q30%**	**GC%**
dsApSOCS-2+Micrococcus luteus	6,923,641,600	23,130,124	18,504,665	80.00	98.10	94.43	42.55
dsApSOCS-2+Micrococcus luteus	7,797,146,398	26,102,106	20,111,717	77.05	98.22	94.63	43.40
dsApSOCS-2+Micrococcus luteus	5,967,163,040	19,942,159	16,024,304	80.35	98.23	94.69	42.91
dsGFP+*Micrococcus luteus*	6,737,393,200	22,531,058	17,926,445	79.56	97.63	93.25	42.68
dsGFP+*Micrococcus luteus*	5,970,481,000	19,948,172	15,703,121	78.72	97.56	93.08	43.35
dsGFP+*Micrococcus luteus*	8,441,303,376	28,210,831	22,130,524	78.45	97.47	92.88	43.35
